# Clinical-pathological study on β-APP, IL-1β, GFAP, NFL, Spectrin II, 8OHdG, TUNEL, miR-21, miR-16, miR-92 expressions to verify DAI-diagnosis, grade and prognosis

**DOI:** 10.1038/s41598-018-20699-1

**Published:** 2018-02-05

**Authors:** Enrica Pinchi, Alessandro Frati, Luigi Cipolloni, Mariarosaria Aromatario, Vittorio Gatto, Raffaele La Russa, Alessandro Pesce, Alessandro Santurro, Flavia Fraschetti, Paola Frati, Vittorio Fineschi

**Affiliations:** 1 Department of Anatomical, Histological, Forensic and Orthopaedic Sciences (SAIMLAL), Sapienza University of Rome, Viale Regina Elena 336, 00185 Rome, Italy; 2 Department of Neurosciences, Mental Health and Sensory Organs (NESMOS), Sapienza University of Rome, Via di Grottarossa 1035, 00189 Rome, Italy; 3 IRCCS Neuromed, Via Atinense 18, 86077 Pozzilli, Italy

## Abstract

Traumatic brain injury (TBI) is one of the most important death and disability cause, involving substantial costs, also in economic terms, when considering the young age of the involved subject. Aim of this paper is to report a series of patients treated at our institutions, to verify neurological results at six months or survival; in fatal cases we searched for βAPP, GFAP, IL-1β, NFL, Spectrin II, TUNEL and miR-21, miR-16, and miR-92 expressions in brain samples, to verify DAI diagnosis and grade as strong predictor of survival and inflammatory response. Concentrations of 8OHdG as measurement of oxidative stress was performed. Immunoreaction of β-APP, IL-1β, GFAP, NFL, Spectrin II and 8OHdG were significantly increased in the TBI group with respect to control group subjects. Cell apoptosis, measured by TUNEL assay, were significantly higher in the study group than control cases. Results indicated that miR-21, miR-92 and miR-16 have a high predictive power in discriminating trauma brain cases from controls and could represent promising biomarkers as strong predictor of survival, and for the diagnosis of postmortem traumatic brain injury.

## Introduction

Traumatic brain injury (TBI) is a group of complex lesions involving the brain and able to determine an alteration in the normal brain function, that occurs as a result of a shock, a blow or a shock or following a head penetrating trauma^1^. In this definition are therefore some conditions that have a different timing of onset (acute or chronic), such as to include under the term TBI different conditions as brain concussion, chronic traumatic encephalopathy, diffuse axonal injury. Each of this conditions occupies a prominent role both in the causes of death statistics, and in physical and mental disability statistics.

It is often difficult discriminate type of damage since the artefacts found at macroscopic and microscopic examination because are often overlapping, in some cases non-specific as it is often possible to find an overlap of pathogenetic mechanisms that do not allow or make it very difficult to distinguish between the primary damage due to trauma and secondary consequences of the same^2^.

Clinical classification of TBI takes into account symptoms severity, evaluated with GCS score^3^, so for which may be divided into:

- Mild TBI when the GCS is between 13 and 15, there has been a significant retrograde amnesia (<1 day), loss of consciousness was short (<30 minutes);
- Moderate TBI when GCS is between 9 and 12, 1–7 days of retrograde amnesia, loss of consciousness lasts between 30 minutes and 24 hours;
- Severe TBI when GCS is between 3–8, retrograde amnesia more than 7 days and loss of consciousness > 24 h.

One of the simplest classification takes in consideration trauma extension that can have a focal trauma (tearing or bruising, haemorrhage, secondary focal lesions with increased intracranial pressure), multifocal or diffuse involvement (DAI, global ischemia, diffuse cerebral edema)^4^.

Another forensic classification divides TBI into:

- Impact injuries are invariably associated with situations where the head hurt with an object;
- Acceleration–deceleration brain injury resulting from unrestricted movement of the head;
- Compressive strains or penetranting injuries when an object passes through the skull and causing directly parenchymal injury.

TBI have a significant health economic relevance, because in spite of a slight variation in incidence rates in different countries, it is believed interests around 200–300 over 100,000 people^5^.

In the USA, CDC has indeed estimated that about 50,000 deaths each year are as a direct consequence of TBI, and a much larger number of individuals develop a more or less severe disabilities^6^.

In Europe the incidence rate is almost the same between each European country, and it is estimated that approximately 7 million people have developed a disability as a result of TBI^3,7^.

The high incidence of TBI and considerable repercussions on the mortality rates of disability, is an important issue in social, health and economics fields^8–17^.

Traumatic brain injury, despite covering different pathological forms, can be traced into two different pathogenetic pathways though partly overlapping each other and fall into two basic underlying mechanisms: a primary damage that is a consequence of destruction of brain tissue with possible axonal shearing and a secondary damage that is a consequence of activation of cellular and molecular pathways such as apoptosis, inflammation and disruption of Blood Brain Barrier (BBB) that can be followed by vasogenic or extracellular edema^18,19^.

Axonal damage can be a consequence of collapse and stretching of the axonal plasma membrane resulting in major dysregulation of ionic transcellular exchange with neuronal depolarization and increased pump activities^20^. Simultaneous activation of multiple intracellular signaling pathways lead activation of immediate transcriptional factors (c-fos, c-jun), that regulate expression of a variety of target genes (growth factors, cytoskeletal proteins, heat-shock proteins (HSPs)^21,22^.

The structural features of DAI were defined by Adams *et al*. using a series of 45 fatal cases^23^.

In its most severe form, DAI has three distinctive structural features:

1. Diffuse supratentorial damage to axons (grade I)
2. A focal lesion in the corpus callosum (grade II)
3. A focal lesion or lesions in the rostral brain stem (grade III)^24^.

Traumatic insult determines in the first instance a considerable increase in the levels of mediators capable of exerting an action on excitotoxicity on neuronal structures such as glutamate. Increased production of free radicals highly reactive can also damage DNA, in addition to direct neurotoxic action due to lipoperoxidation and consequent neuronal membrane damage^25^.

Aim of this paper is to report a series of patients treated at our institutions, to verify neurological results at six months or survival; in fatal cases we searched for β-APP, IL-1β, GFAP, NFL, Spectrin II, 8OHdG, TUNEL and miR-21, miR-16, and miR-92 expressions in brain samples, to verify DAI diagnosis, and grade as strong predictor of survival and inflammatory response. We searched immunohistochemical expression of 8-hydroxy-2-deoxyguanosine (8OHdG) which is an oxidized derivative of deoxyguanosine. 8-Oxo-dG is one of the major products of DNA oxidation. Concentrations of 8OHdG within a cell are a measurement of oxidative stress^26^. The selection of miRNAs candidates was made by searching in available literature for traumatic brain injury-specific miRNAs which should also be expressed post mortem. The vast majority of scientific reports dealing with traumatic brain injury employs murine models^27,28^. Concerning studies on humans, most of them are based on cell lines or biological fluids, such as plasma and serum^29,30^. On the contrary, our work deals with paraffin-embedded brain sections.

To our knowledge, this is the first experimental study documenting the differently expressions of miRNAs in paraffin-embedded sections of subjects with complex traumatic brain injury with respect to controls.

## Results

**Figure 1:**
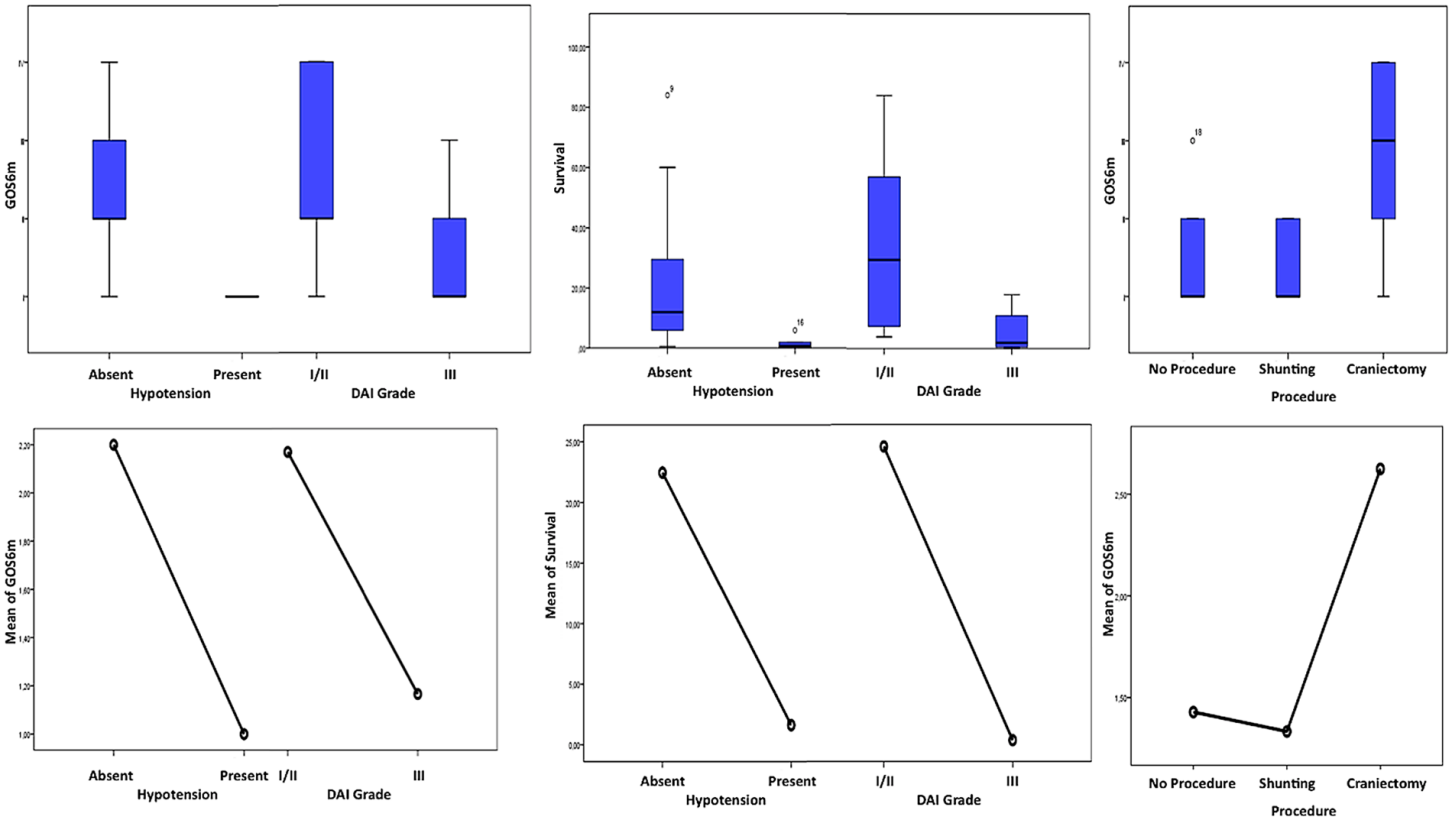
Impact of DAI grading, hypotension and surgery on GOS. Impact of DAI grading and hypothension on (**A-A1**) GOS at 6 months and (**B-B1**) survival. Impact of surgery on (**C-C1**) GOS at 6 months.

Sex did not affect neurological results at six months (GOS) or survival. DAI grade was a strong predictor of survival and presented an association with GOS at six months (p.002; p.065 respectively) (Fig. 1A-A1). The specific mechanism of trauma did not affect GOS or survival. Similarly, the multiple trauma in place of a predominantly head trauma did not predict, in this cohort, a worse neurological outcome or survival. The diagnosis of MOF was a predictor of lesser survival and worse GOS outcome (p.018; p.049 respectively); uncontrolled hypotension disclosed similar statistical association (p.010; p.059 respectively) (Fig. 1A-A1,B-B1).

**Figure 2:**
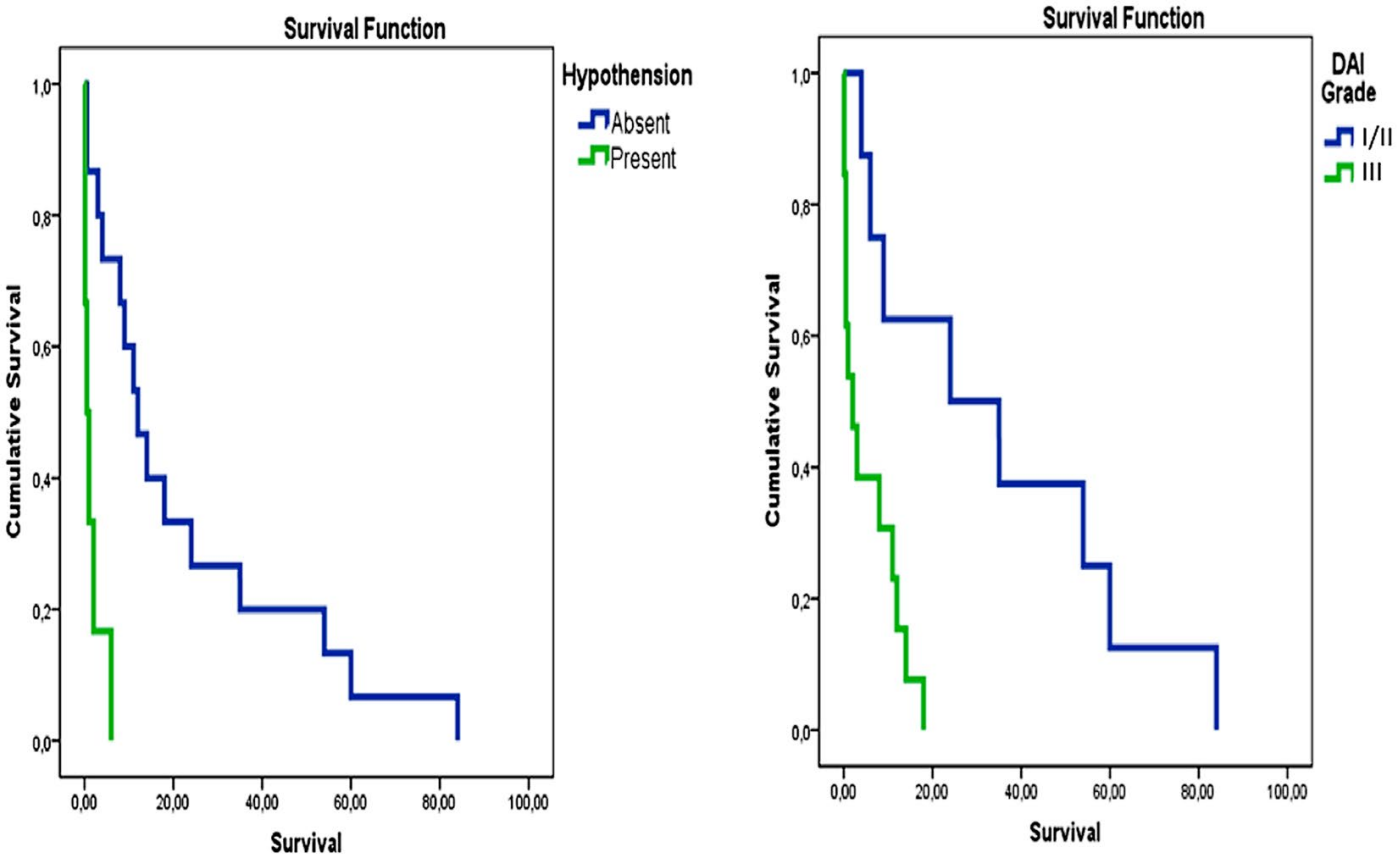
Kaplan-Meier analysis, comparing DAI and hypothension subgroups. The analysis confirms advantage for the patients suffering from a lower DAI grading and not experiencing uncontrolled hypotension.

Decompressive craniectomy had a significant impact on GOS at 6 months (p.015) in respect to conservative treatment or just ventriculostomy, but not on survival (Fig. 1C-C1). We furthermore performed a Kaplan-Meier analysis to retrieve survival differences among the different subgroups (Fig. 2). Such analysis completely confirmed the survival advantage for the patients suffering from a lower DAI grading and not experiencing uncontrolled hypotension.

The histological and immunohistochemical characteristics of the study group compared to the control group are shown in Table 1.

**Table 1:** Statistical analysis of the immunohistochemical findings and gradation of the immunohistochemical reactions. Responses about antibody anti-β-APP, IL-1β, anti-GFAP, anti-NFL, anti-Spectrin II, anti-8OHdG and TUNEL assay expressions in brain specimens (thalamus, hypothalamus and striatum).

	Control group	TBI group	Thalamus	Hypothalamus	Striatum	Statistical value TBI vs Control
anti-β-APP	+/−	+++	+++	+++	+++	*** p < 0.001
IL-1β	−	+++	+++	+++	+++	*** p < 0.001
Anti-GFAP	+/−	+++	+++	+++	+++	*** p < 0.001
TUNEL assay	+/−	+++	+++	+++	+++	*** p < 0.001
Anti-NFL	+	+++	+++	+++	+++	*** p < 0.001
Anti-Spectrin II	+/−	+++	+++	+++	+++	*** p < 0.001
Anti-8OHdG	+/−	+++	+++	+++	+++	*** p < 0.001

NS: p > 0.05; * p < 0.05; ** p < 0.01; *** p < 0.001. Intensity of immunopositivity was assessed semiquantitatively in the scale 0–4 as follows: −: no immunoreactivity (0%); +: mild immunopositivity in scattered cells (10%); ++: immunopositivity in up to one third of cells (33%); +++: immunopositivity in up to two- third of cells (70%) and ++++: strong immunopositivity in the majority or all cells (100%). In cases of divergent scoring, a third observer decided the final category.

**Figure 3:**
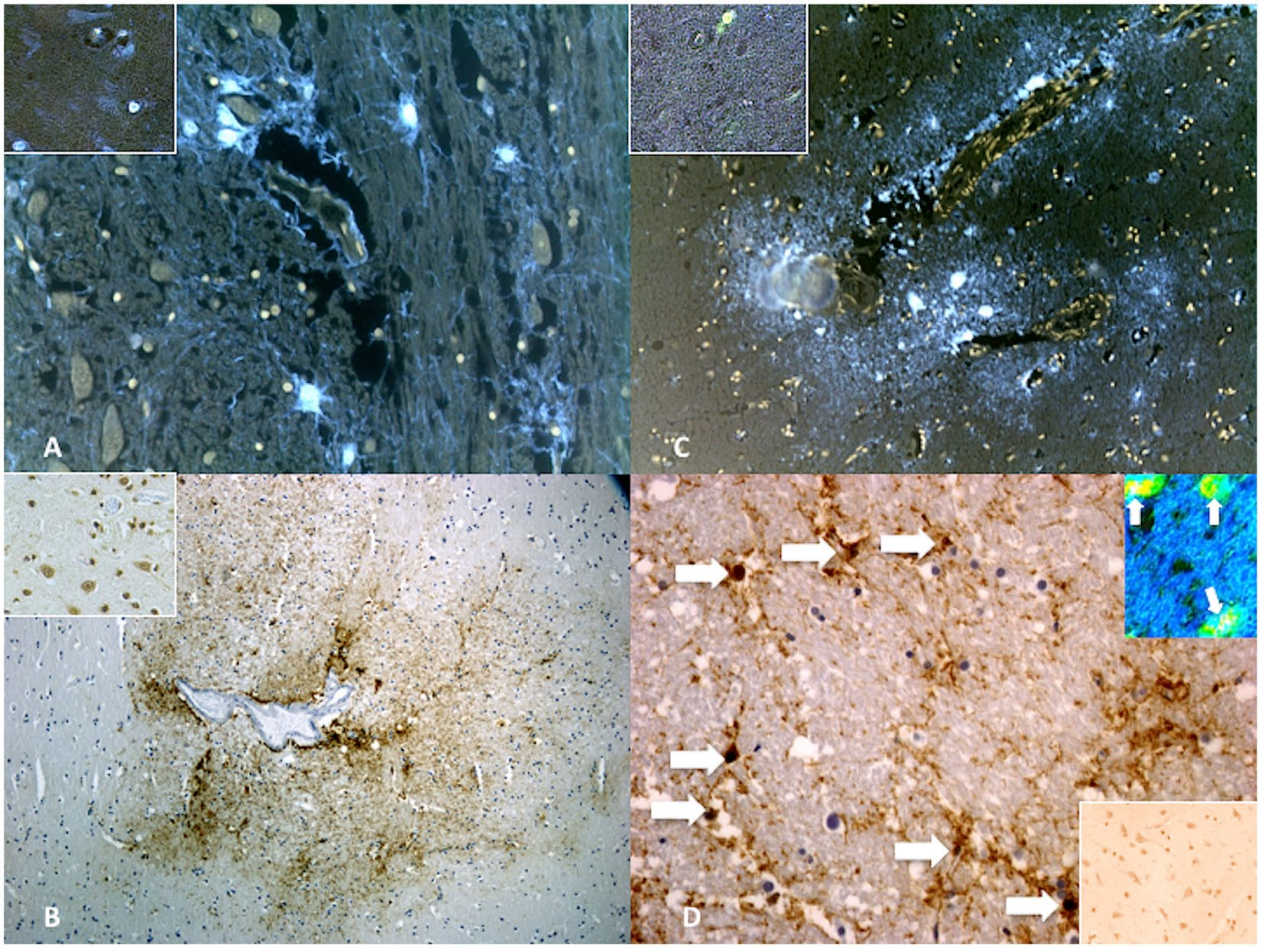
Immunohistochemical reactions for anti-β-APP, anti-IL-1β, anti-GFAP and TUNEL assay. (**A**) Anti-β-APP reaction was prominent in the axonal bulbs and fibers of the affected regions in the TBI group. Insert represents normal reactions from control group. (**B**) Reaction to IL-1β was intensely positive mainly in neurons. Insert represents normal reactions from control group. (**C**) In acute cases a diffuse anti-GFAP reaction was evident in sporadic fields. Insert represents normal reactions from control group. (**D**) The apoptotic nuclei (arrows) appeared isolated or irregularly distributed over the entire brain section. (Insert) Confocal laser scanning microscope: typical morphological features of neuronal (green) apoptosis (arrows) associated with marked condensation of chromatin and its fragmentation into discrete bodies (yellow). Insert represents normal reactions from control group.

Semi-quantitative evaluation (grade 0–4) revealed increased immunoreaction of β-APP in TBI cases (Fig. 3A). GFAP expression was significantly increased in the TBI group with respect to control group subjects whose death was from other causes (Fig. 3B). Intense IL-1β immunoreaction in the TBI group matched by the negative marker of expression in the control group (Fig. 3C). Apoptosis was measured by TUNEL assay and values of cell apoptosis in the study group were significantly higher than control cases. The apoptotic nuclei appeared isolated or irregularly distributed over the entire brain section. The percentage of apoptotic nuclei was determined. The immunohistochemical study revealed an intensive positive result to TUNEL assay (Fig. 3D): 60 ± 13% and 7 ± 1% apoptotic cells were observed in TBI group and control group, respectively.

**Figure 4:**
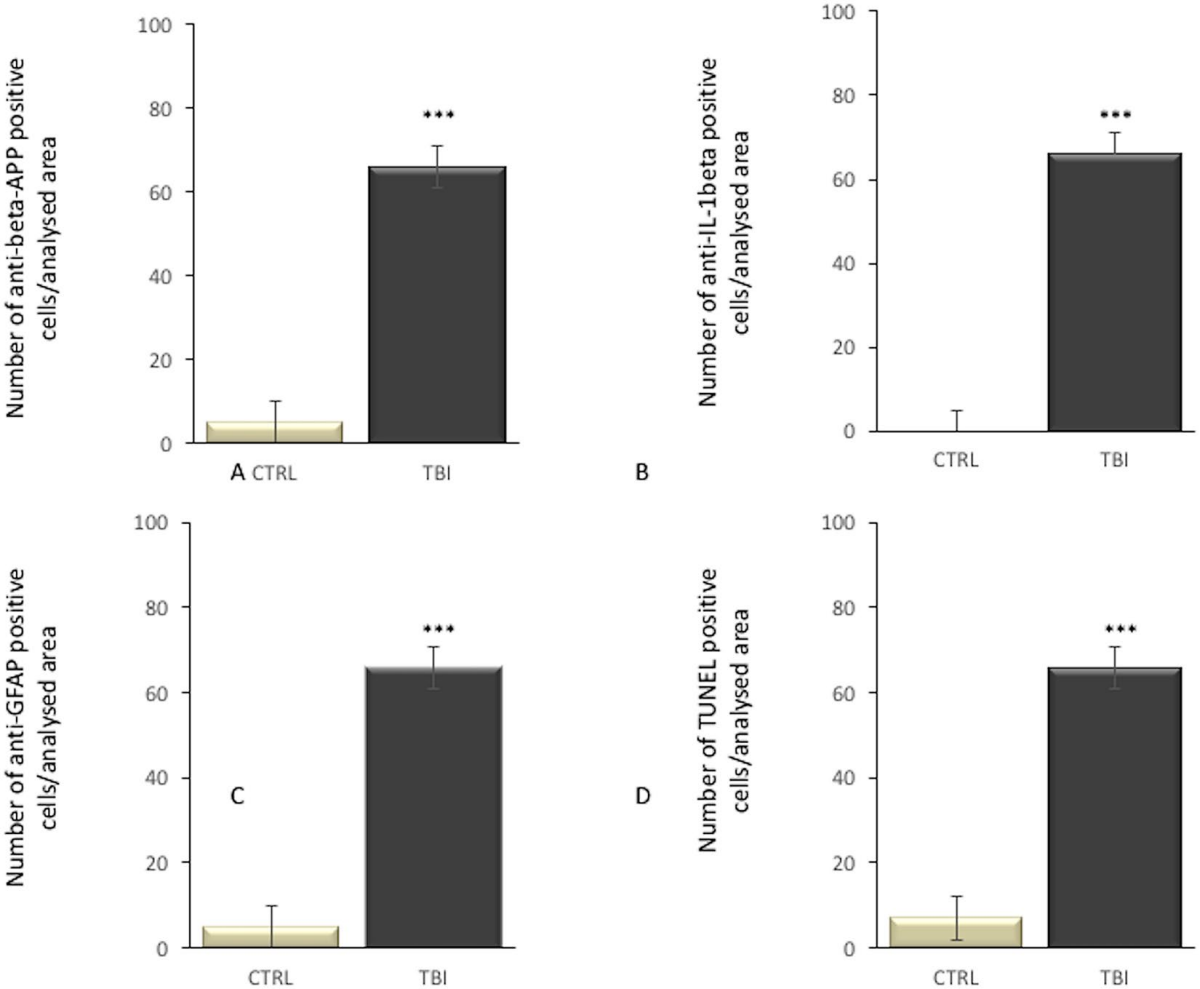
Statistical analysis of the immunohistochemical findings and gradation of the immunohistochemical reactions. Responses about antibody (**A**) anti-β-APP, (**B**) IL-1β, (**C**) anti-GFAP and (**D**) TUNEL assay expressions in brain specimens (thalamus, hypothalamus and striatum).

Statistical analysis of the immunohistochemical findings and gradation of the immunohistochemical reactions are summarized in Fig. 4.

**Figure 5:**
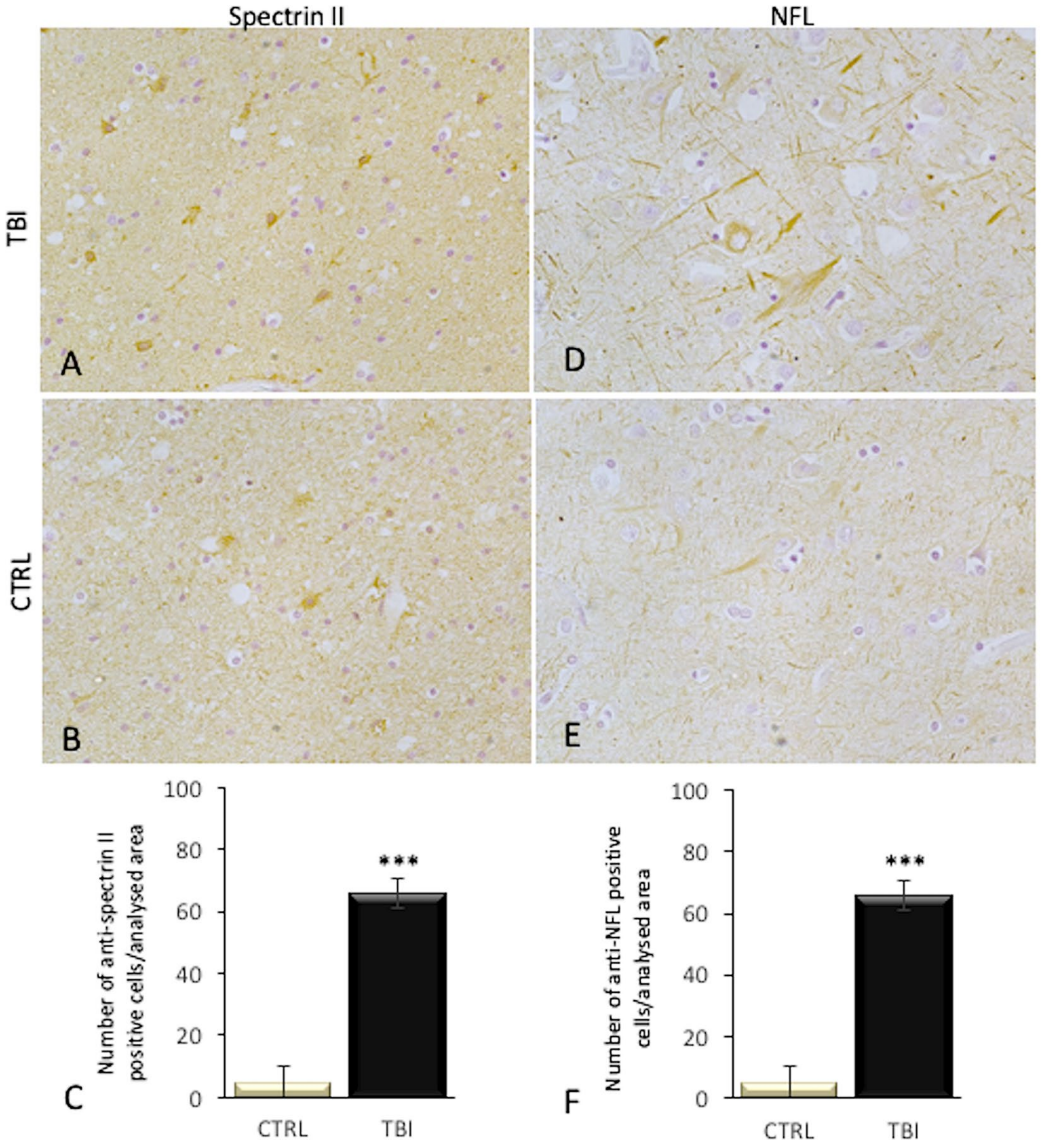
Immunohistochemical reactions for anti-spectrin II and NFL with statistical analysis of the immunohistochemical findings and gradation of the reactions. (**A**) Anti-spectrin II reaction was intensely positive mainly in TBI neurons, compared to control group (**B**), as reported (**C**). NFL expression was significantly increased in the TBI group (**D**) with respect to control group (**E**), as confirmed in statistical analysis (**F**).

**Figure 6:**
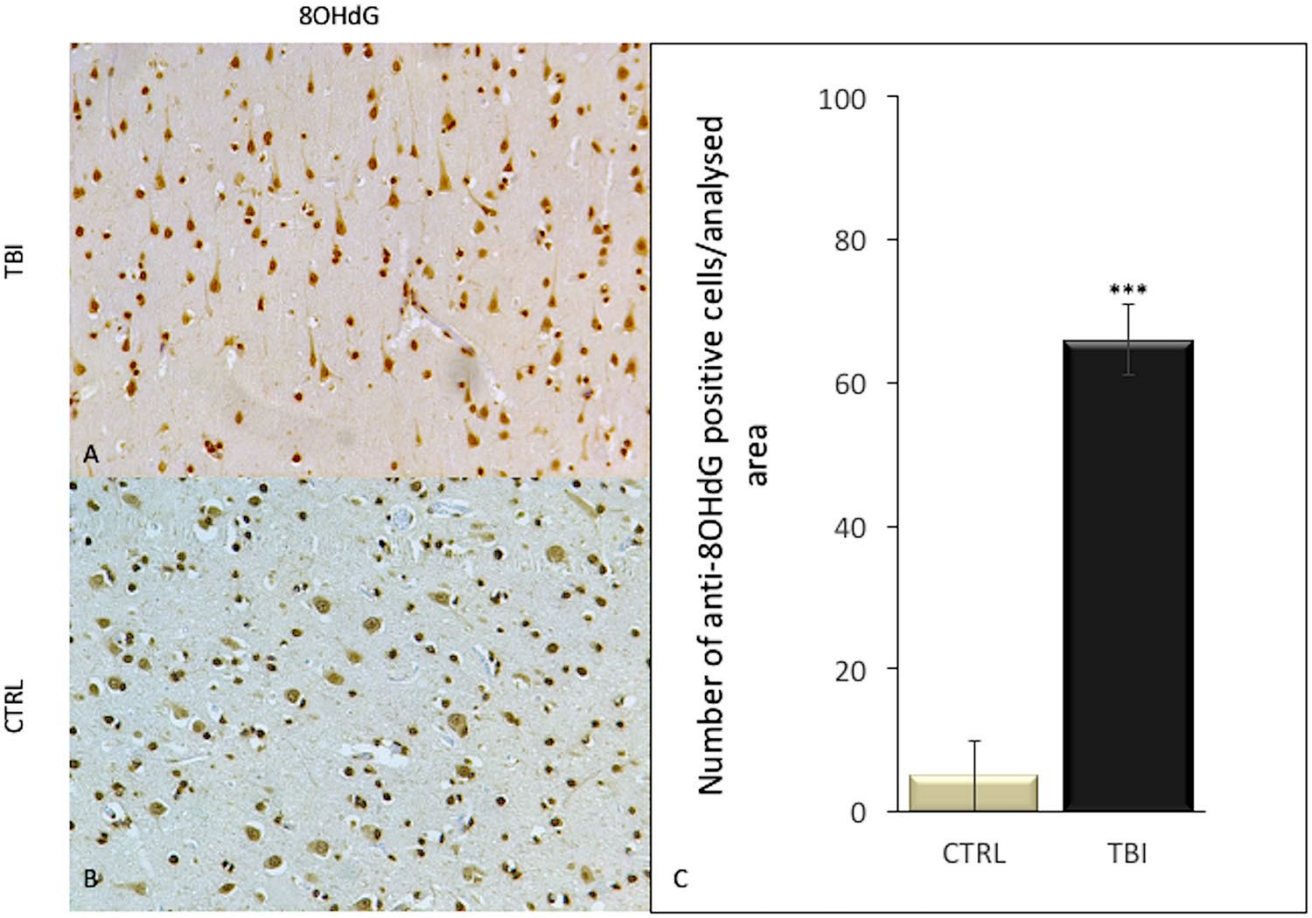
Immunohistochemical reactions for anti-8OHdG with statistical analysis of the immunohistochemical findings and gradation of the reactions. (**A**) Anti-8OHdG reaction was intensely positive mainly in TBI neurons, compared to control group (**B**), as confirmed in statistical analysis (**C**).

Also anti-spectrin II and NFL reactions were intensely positive mainly in TBI neurons, compared to control group (Fig. 5). While 8OHdG immunoreactivity was detected in very few cells of the frontal cortex of control group, a significant elevation of the number of 8OHdG positive cells was observed in subjects died following TBI (Fig. 6).

**Figure 7:**
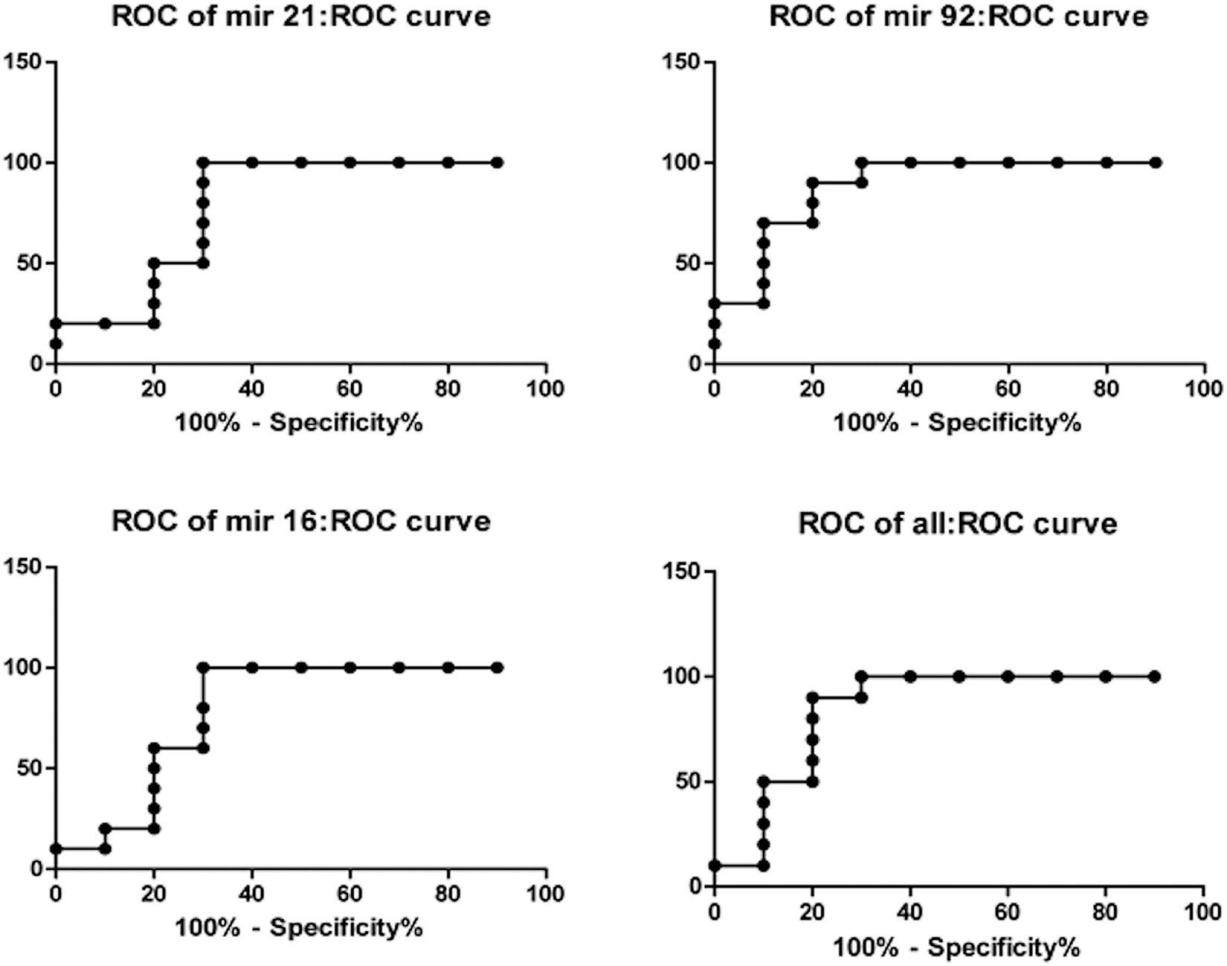
ROC and AUCs for TBI group and control group. Receiver-operating characteristic (ROC) curves were built and the areas under the curve (AUCs) were calculated for each miRNA differently expressed in brain sections of subjects who died for traumatic brain injury and control group.

To assess the ability of each miRNA to discriminate between samples and controls, receiver-operating characteristic (ROC) curves were built and the areas under the curve (AUCs) were calculated (Fig. 7A–D). Univariate analysis for each miRNA led an AUC of 0.79 (95% CI = 0.57–1.01, p = 0.03) for miR-21 (Fig. 7A), an AUC of 0.79 (95% CI = 0,57–1,01 p = 0.029) for miR-16 (Fig. 7B) and AUC of 0.89 (95% CI = 0.73–1.04 p = 0.003) for miR-92 (Fig. 7C). A multivariate analysis was performed combining the three miRNAs and gave an AUC of 0.85 (95% CI = 0.66–1,04, p = 0.008) (Fig. 7D).

**Figure 8:**
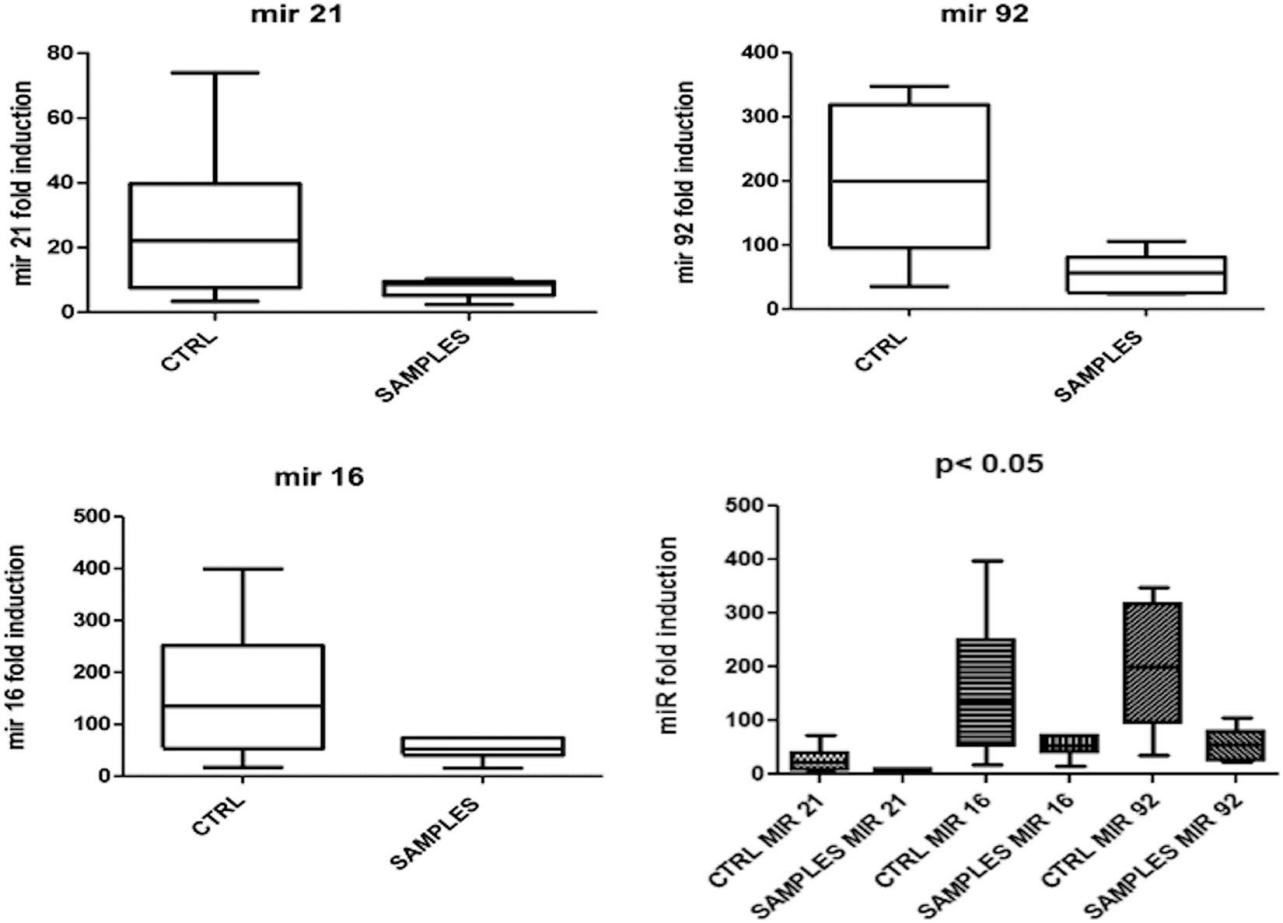
Expression of miR-21, miR-16, and miR-92. Expression of these miRNA was significantly higher in controls than subjects who died for traumatic brain injury.

These data indicate that these three miRNAs have a high predictive power in discriminating trauma brain cases from controls and could represent promising biomarkers for the diagnosis of postmortem traumatic brain injury. In particular, the expression of miR-21 was three times higher in controls than subjects who died for traumatic brain injury (Fig. 8).

## Discussion

Pathological diagnosis of brain injury can be difficult in some cases or can leave diagnostic doubts, especially in cases of mild given, or in cases when instrumental examinations are negative, or macroscopic examination does not always allow to appreciate evident alterations, as in the case of DAI where the diagnosis is today possible only through in-depth instrumental analysis.

For these reasons the search for blood markers that can assist clinicians in diagnosis and staging of TBI and immunohistochemical markers that can help pathologist in evaluation of traumatic brain injury and survival time interval estimation has always aroused considerable interest.

In the present work, we searched for a panel of selected traumatic brain injury-specific miRNAs differently expressed in brain sections of subjects who died for traumatic brain injury and control group. The selection of miRNAs candidates was made by searching in available literature for traumatic injury-specific miRNAs which should also be expressed post mortem. The vast majority of scientific reports dealing with traumatic brain injury employs murine models^27,28^. Concerning studies on humans, most of them are based on cell lines or biological fluids, such as plasma and serum^29–31^. On the contrary, our work deals with paraffin-embedded brain sections. To our knowledge, this is the first report documenting the downregulation of miR-21 in paraffin-embedded sections of subjects with complex traumatic brain injury with respect to controls. In particular, the expression of miR-21 was three times higher in controls than subjects who died for traumatic brain injury.

However, several miRNA profiling studies using biological and functional approaches on TBI were performed^32–36^ to dissect the role miRNAs significantly altered in TBI. Concerning *in vitro* studies, Bhalala *et al*. reported the increased miR-21 expression following spinal cord injury correlate with astrocyte hypertrophy, which represents an event associated with TBI^37^. These facts, together with the evidence that miR-21 is able to promote axon growth, led researchers to hypothesize a role for miR-21 as a neuroprotective molecule in TBI^38,39^. Another study on murine astrocytes and neuronal cells reported temporal differences in a pattern of miRNAs after ischemia: miR-21 was upregulated at 12 h and at 24 h post-OGD (oxygen-glucose deprivation) as compared to expression in control neurons. In astrocytes undergoing OGD, miR-21 levels increased only 12 h after OGD (1.6 > fold, p < 0.05)^27^. Similarly, Ge and colleagues found an increased expression of miR-21 in rat brains after traumatic brain injury (TBI). They hypothesized this could protect against blood-brain barrier (BBB) damage, inhibiting neuronal apoptosis and promoting angiogenesis^40^. So far, the overexpression of miR-21 has been described in biological fluids from subjects with brain injury. For example, a recent study by Illerhaus and Batchelor found elevated levels of miR-21 in cerebrospinal fluids from 23 primary CNS lymphoma (PCNSL) patients compared to 30 controls^41^.

An increase in basal expression of miR-21 was observed in aged mice brain compared to adult counterpart. The increase in miR-21 levels following TBI has been reported in the adult mice, with a maximum peak 24 hours after injury. On the contrary, miR-21 levels decreased after TBI in the aged mice^42^. Furthermore, a downregulation in miR-21 levels has been reported by Nelson *et al*. on snap-frozen white versus grey matter tissue from Alzheimer’s patients^43^. It is worth noting that a proper comparison between our and other reports cannot be made, in terms of starting material and experimental conditions. Nonetheless, similarly to what has been said for miR-21, miR-92 and miR-16, in our cases we observed a decrease in expression in traumatic brain injury cases with respect to controls. In fact, their expression in controls was three times higher than subjects who died for traumatic brain injury. In accordance with our results, a study performed by Redell *et al*. explored plasma miRNA profiles in severe traumatic brain injury patients and healthy volunteers and found decreased levels of miR-16 and miR-92, which were demonstrated to behave as good markers of severe TBI at 25–48 h after injury^44^.

These data indicate that these three miRNAs have a high predictive power in discriminating trauma brain cases from controls and could represent promising biomarkers for the diagnosis of postmortem traumatic brain injury^45^.

A field of very promising research in evaluation of brain injury, in addition to the identification of possible blood markers useful for the assessment and clinical staging of TBI, is the search for newer immunohistochemical markers that may be useful to the pathologist for timing of traumatic brain injury. In fact, currently used markers do not always allow to discriminate acute TBI from a sub-acute (<8 h after the traumatic event), because lies in the lack of sensitivity in the acute phase if survival time is less of 12 hours for the HE preparation and for detection of β-APP with a time that is more of 12 h for silver preparation or CD68. Actually, the main immunohistochemical markers used for the evaluation of traumatic brain injury include β-APP (β-amyloid precursor) and anti-glial fibrillary acid protein (GFAP)^46,47^.

β-APP is a surface protein that has multiple functions and play an important role in many neuronal intracellular processes^48^ and can be considered one of the most reliable marker of a diffuse axonal injury. The β-APP has proven to be one of the most selective immunohistochemical markers in identifying and diagnosing a diffuse axonal injury^49,50^. In fact, the positive immunohistochemistry for β-APP is not detectable in undamaged brain tissue samples, while there has been an increase in immunoreactivity from 2 h after a traumatic brain injury^51,52^.

More recent studies have also enabled to detect how it is possible to highlight a different immunohistochemical patterns in β-APP immunoreactivity which may aid in discriminating a traumatic brain injury from one type of ischemic type^53^.

The immunoreactivity for GFAP and S100 in astrocytic level can be used for the timing of the brain injury having been shown that such positivity is significantly lower in subacute cases, or delayed and having Li *et al*. also describes a possible correlation between the severity of trauma and the immunopositivity for GFAP and S100^54^.

Li *et al*. have shown that, in rats, serum and CSF levels of GFAP can be considered as potential markers in severe or moderate cases of traumatic brain injury, also highlighting a positive correlation between the linear acceleration and power of the trauma with serum and CSF levels of these markers.

However GFAP seems to be a better biomarker of mild TBI than is S100-B, because has not been detected extracerebral expression of GFAP. Li suggest that GFAP-immunopositivity showed that subacute/delayed death cases with a survival period over 12 h after brain injury showed lower astrocyte GFAP-immunopositivity in Hyppocampal CA4 region.

Neurofilaments are the main cytoskeletal components in neuronal cells, important for maintaining axonal caliber. After axonal cell injury caused by direct trauma increased levels of light neurofilament were observed^55^.

Spectrin plays important roles in nervous cells, it exhibited periodic structures alternating with those of actin and adducin, and the distance between adjacent actin-adducin rings was comparable to the length of a spectrin tetramer^56,57^.

Among the potentially most promising marker for the timing in the acute phase of brain injury covered oxidative stress markers^58–61^. In this study, we investigated the possible contribution of 8OHdG one of the predominant form of ROS-induced oxidative damage to DNA, which has therefore been widely used as a very reliable biomarker for oxidative stress presence. We found that expression of 8OHdG was significantly increased in the group of subjects died following TBI, with respect to subjects from control group.

Most of alterations induced by TBI are related with oxidative stress and how widely demonstrated play a very important role on TBI sequelae. Oxidative stress is implicated on the pathogenesis of primary damage but also have a significant role in secondary injuries evolution^62,63^.

Oxidative stress, due to increased production of neurotoxic amino acids action, on all the glutamate, in fact increases in production of free radicals with a important alteration of neuronal homeostasis^64–66^. Traumatic insult determines in the first instance a considerable increase in the levels of mediators capable of exerting an action on excitotoxicity on neuronal structures such as glutamate. This increase is a result of increased release by the damaged cells and as consequence of the increased activities of the astrocytic glutamate transporter. The increased activation of glutamate receptors causes an increase of intracellular concentration of calcium that is responsible for the induction of different biosynthetic pathways, including NOS pathway. The increased production of NO, contextually to high concentration of intracellular of calcium, play a toxic action on mitochondrial function, that results in an increased activation of cathepsin with induction of apoptotic pathways. Increased production of free radicals highly reactive can also damage DNA, in addition to direct neurotoxic action due to lipoperoxidation and consequent neuronal membrane damage^25^.

Immunohystochemical studies have shown that oxidative stress secondary to trauma leads to increased exposure of the neuronal structures and especially hippocampal cytokines. This increased exposure is reflected on the activity of glial cells (astroglia and microglia) that mediate the response to traumatic damage. Turyn *et al*. have shown that there are early time-dependent changes in cellular oxidative markers (GSSG, protein carbonyls, 3-NT, 4-HNE, and acrolein), the antioxidant system (GSH, GST, GPx, GR, G-6PD, Cu/Zn- or Mn-SOD, and CAT), and synaptic proteins (synapsin I, PSD-95, and SAP-97) in the adult rat hippocampus following experimental TBI^67^.

Frugier *et al*. analyzed humoral and cellular inflammation elicited after TBI in post mortem human brain tissue and found that pro-inflammatory cytokine protein levels of IL-1β, IL-2, IL-6, IL-8, IFN, TNF-α and GM-CSF were significantly increased after an acute brain injury in humans. Same authors have produced a quantitative analysis of mRNA levels of several inflammatory mediators, and have found a significantly increased following TBI^66^.

Our results showed that the reaction to IL-1β, although intense, was positive mainly in neurons since the acute stages and in neurons but also in glial cells in the following stage (non-acute). In particular, cytokine IL-1β positive reaction was observed in neurons only in non-acute cases, associated to vascular and glial positivity. The expression in acute cases was shown mainly at vascular level.

Traumatic brain injury often produces neuronal apoptosis as direct effect of trauma or as a consequence of hypoxic changes, for increased intracranial pressure or oedema.

Recent studies have shown that apoptosis contributes to neuronal and glial cell loss following both acute neurological disorders and in traumatic brain injuries (TBI) lead to activation of caspases’s pathways, where caspase-3 play a very important role.

Tao *et al*. have found that follow a TBI a caspase 3 reaction was detectable in cortical neurons after a post-traumatic interval of 80 min from trauma, with a peak level reached after 48 h. The expression of caspase-3 increased after TBI, reached a peak 24–48 h after TBI, and then decreased. The present results suggest that the determination of TUNEL expression could hardly be used to estimate TBI time course unless morphological changes of positive cells been considered^68^.

## Conclusions

Proper survival of the clinical course of the Brain Traumatic Injury today still represents a great challenge for the clinicians, the considerable implications that proper dating takes in assessing the clinical case. For the pathologist, the historical immunohistochemical markers used (β-APP, GFAP) will continue to play an important role in the diagnostic evaluation process; the best understanding of the molecular mechanisms involved in the pathogenesis of the resulting damage to a TBI, however, and the understanding of oxidative stress leading to apoptosis will assume greater importance, as the potential to allow for a more precise diagnosis in time compared to classic markers. Difficulty in the use of infiammatory markers such as immunohystochemical markers of TBI in early stage, is due to the overlap of secondary changes after traumatic event (es. hypoxia, edema) that can lead to difficult to errors in estimating the survival time. Our data indicate that miR-21, miR-92 and miR-16 have a high predictive power in discriminating trauma brain cases from controls and could represent promising biomarkers as strong predictor of survival, and usefulness tool for postmortem diagnosis of traumatic brain injury.

## Materials and Methods

### Clinical study

Between June 2008 and June 2016 a total of 21 patient suffering from severe TBI were admitted at Sant’Andrea Emergency Department - Sapienza University of Rome, and subsequently clinically diagnosed with DAI. Diagnosis was performed according to the following clinical and radiological criteria: a coma lasting more than 6 hours after a TBI with no space occupying hemorrhagic or ischemic lesion or focal swelling on CT scan. In the final cohort, a total of 18 males and 3 females were included.

When clinical conditions were judged to be completely stable, patients underwent elective brain MRI scan. On the ground of this MRI scan each patient was classified according to the classification proposed by Gennarelli, based on the topographical distribution of the lesions^69^. A total of 7 patients were diagnosed with DAI grade II, 1 patient grade I, while a total of 13 patients presented radiological signs of brainstem involvement and therefore were classified as grade III. The most common cause of incident was car accident, we recorded 6 motorcycle accidents, 2 precipitated and 2 ran over patients and one patient victim of violence. Sixteen of these patients suffered from a multiple trauma rather than just a head trauma.

The average GCS at admission was 4,75 ± 1,64. Physicians of the Post-Acute Rehabilitation centers and families were contacted by phone call, during December 2016, to undergo a simple phone questionnaire inspired to Glasgow Outcome Scale (GOS). A total of 10 patients (47,6% of the total) were dead at six months after trauma. An adjunctive mortality of 14,28% was recorded at 12 months after trauma. A total of 5 patients (23,08%) were alive. Therefore, the average GOS at 6 months, among the survived patients, was 2.54 and overall average survival was 16.5 ± 23.07 months.

A total of 14 patients underwent a surgical procedure of ventriculostomy, external shunting. In all of these patients the catheter had an ICP sensor in order to monitor intracranial pressure. In 8 of these patients, intensive care physicians were not able to control ICP conservatively and underwent bilateral craniectomy. ICP value higher than 20mmH_2_O not responsive to conservative treatments was considered as a surgical indication to perform bilateral decompressive craniectomy.

In 7 patients, at admission GCS was 3, were hemodynamically unstable, pupillary light reflex was obviously torpid or patient was midriatic, brain CT scan disclosed obvious brainstem hypodensity and no surgical procedure was performed.

During the first 72 hours of ICU stay, a total of 6 patients were diagnosed with severe hypotension (hypotension was considered as systolic blood pressure under 90 mmHg despite intravenous injection of inotropic agents). Multiple Organ Failure, as a result of systemic inflammation caused by severe infections was recorded in the 3 months after trauma in 7 patients (33,3%) (Table 2).

**Table 2:** Clinical data of 21 patient suffering from severe TBI, and subsequently clinically diagnosed with DAI.

Case	Sex	Age	DAI grade (MRI scan)	GCS at admission	GOS at 6 months	Type of incident	Multiple or head trauma	Surgical procedure	MOF within 2 months	Hypotension	Survival (months)	Congo red, anti-β-APP, GFAP, IL-1β, NFL, Spectrin II, 8OHdG, TUNEL assay	miR-21, miR-92, miR-16
1	M	52	2	7	3	Moto accident	Mainly Head	Craniectomy	No	No	Alive (35)		
2	M	16	2	6	2	Car accident	Multiple Trauma	Shunting	No	No	Alive (54)		
3	M	28	2	5	2	Violence	Multiple Trauma	Craniectomy	No	No	9	+	+
4	M	39	3	5	2	Car accident	Multiple Trauma	Craniectomy	Yes	No	14	+	+
5	M	34	2	8	1	Car accident	Mainly Head	Shunting	No	No	4	+	+
6	F	54	3	3	1	Car accident	Multiple Trauma	No	Yes	Yes	0.1	+	+
7	M	25	3	4	1	Moto accident	Multiple Trauma	Shunting	Yes	No	0.5	+	+
8	M	69	3	3	1	Car accident	Multiple Trauma	No	No	Yes	2	+	+
9	M	37	2	4	2	Car accident	Multiple Trauma	No	No	No	Alive (84)		
10	M	46	3	3	2	Ran over	Mainly Head	Shunting	No	No	8	+	+
11	M	50	1	7	4	Car accident	Multiple Trauma	Craniectomy	No	No	Alive (24)		
12	M	72	3	3	1	Precipitation	Mainly Head	No	Yes	Yes	0.1	+	+
13	M	49	3	4	2	Moto accident	Multiple Trauma	Craniectomy	No	No	11	+	+
14	M	31	3	5	3	Ran over	Multiple Trauma	Craniectomy	No	No	18	+	+
15	M	44	3	5	1	Car accident	Multiple Trauma	Shunting	Yes	No	3	+	+
16	F	36	2	3	1	Car accident	Multiple Trauma	No	No	Yes	6	+	+
17	M	56	3	5	1	Precipitation	Multiple Trauma	No	Yes	Yes	0.5	+	+
18	M	25	3	5	3	Moto accident	Multiple Trauma	No	No	No	12	+	+
19	M	32	3	3	1	Car accident	Multiple Trauma	Shunting	Yes	No	0.5	+	+
20	F	19	3	4	1	Moto accident	Multiple Trauma	Craniectomy	No	Yes	1	+	+
21	M	15	2	8	4	Moto accident	Mainly Head	Craniectomy	No	No	Alive (60)		

### Immunohistochemistry study

A total of 26 archived formalin-fixed, paraffin-embedded (FFPE) brain tissue blocks were processed in the Department of Anatomical, Histological, Forensic and Orthopedic Sciences of Sapienza University of Rome. Of these, 16 were cases of TBI enrolled in the present study with fatal outcome, and 10 were control cases (sudden cardiac death). The processing of the data reported in this paper is covered by the general authorization to process personal data for scientific research purposes granted by the Italian Data Protection Authority (1 March 2012 as published in Italy’s Official Journal no. 72 dated 26 March 2012) since the data do not entail any significant personalized impact on data subjects. Our study does not involve the application of experimental protocols; therefore it does not require approval by an institutional and/or licensing committee. In all cases, local prosecutors opened an investigation, ordering that an autopsy be performed to clarify the exact cause of death.

Brain tissues from cases and controls were fixed in 10% buffered formalin for 48 hr. 4 μm-thick paraffin-embedded sections were cut and stained with hematoxylin and eosin. In addition, immunohistochemical investigation of the frontal section with the structures of thalamus, hypothalamus, and striatum was performed with antibodies β-APP (Novocastra, Newcastle upon Tyne, United Kingdom), GFAP (Santa Cruz, CA, USA), IL-1β (Santa Cruz, CA, USA), 8OHdG (1:10, JaICA, Japan), anti-70 kD Neurofilament Light antibody (Abcam, Cambridge, UK), AlphaIISpectrin/SPTAN1 polyclonal antibody (Thermo Fischer, IL, USA) and TUNEL assays (Chemicon, Temecula, CA). 4-μm-thick paraffin sections were mounted on slides covered with 3-amminopropyl-triethoxysilan (Fluka, Buchs, Switzerland). A pretreatment was necessary to facilitate antigen retrieval and to increase membrane permeability to antibodies: antibody anti-β-APP was boiled in 0.1 M citric buffer; antibody anti-IL-1β and antibody anti-GFAP were boiled in 0.25 M EDTA buffer. The primary antibody was applied in a 1:300 ratio for β-APP, in a 1:4000 ratio for IL-1β and in a 1:500 ratio for GFAP. Primary anti-GFAP and anti-IL-1β antibodies were kept at room temperature for 120 min, whereas the incubation of primary anti-β-APP antibody was overnight. For TUNEL assay the sections were covered with the TdT enzyme, diluted in a ratio of 30% in reaction buffer (Apotag Plus Peroxidase *In Situ* Apoptosis Detection Kit; Chemicon) and incubated for 60 min at 38 °C.

Detection was performed with LSAB1 Kit (Dako, Carpinteria, CA), a refined avidin-biotin technique in which a biotinylated secondary antibody reacts with several peroxidase-conjugated streptavidin molecules. The positive reaction was visualized by DAB (3,3-diaminobenzidinetetrahydrochloride hydrate) peroxidation according to standard methods. Then, the sections were counterstained with Mayer’s hematoxylin, dehydrated and coverslipped. Furthermore, retraction balls and amyloid deposits were detected with Congo Red staining kit (Bio – Optica Milano, Italia). All samples were observed under light microscopy (Leica DM4000B optical microscope, Leica, Cambridge, United Kingdom) and confocal microscopy. Finally, a three-dimensional reconstruction was performed (True Confocal Scanner; Leica TCS SPE).

### miRNAs study

#### RNA Extraction

Total RNA, including miRNAs, was extracted from FFPE tissues using the High Pure miRNA isolation kit (Roche Basilea, Switzerland) according to the manufacturer’s instructions. RNA concentrations were evaluated wirh Nanodrop (ThermoScientific Darmstadt, Germany).

#### Reverse Transcription and Quantitative Real-Time PCR

Reverse transcription and quantitative real-time PCRs were performed for the following miRNAs: miR-21, miR-92, miR-16 (Table 3). Each sample was reverse-transcribed with TaqMan MicroRNA Reverse Trascription Kit (Applied Biosystems Darmstadt, Germany) according to the manufacturer’s protocol. Total RNA was converted into cDNA by sequential incubation with reverse transcriptase (RT) at 16 °C for 30 min, 42 °C for 30 min, 85 °C for 5 min and 4 °C. The same amount of RNA (10 ng) for each sample was added to the RT reaction mix for each miRNA of interest in a total volume of 15 μL of RT reaction, as recommended by the manufacturer (Applied Biosystems, Protocol 4367038 Rev. E).

**Table 3:** miRNAs sequences used for reverse transcription and quantitative real-time PCR.

miRNA name	Identification	Accession number	Primers sequence
miR-21-5p	hsa-miR-21	MIMAT0000076	tagcttatcagactgatgttga
miR-92a-3p	hsa-miR-92	MIMAT0000092	tattgcacttgtcccggcctgt
miR-16-5p	hsa-miR-16	MIMAT0000069	tagcagcacgtaaatattggcg

Sequences were reviewed on miRNA database^70,71^ at http://www.mirbase.org.

For PCR assays, TaqMan MiRNA Assay (Applied Biosystems, Darmstadt, Germany) were used. RT-qPCR was performed with Stratagene Mx3005p Real-Time PCR equipment with 96-well optical reaction plates. PCR reaction mixture (total volume 20 μL) contained 1.33 mL of RT product, 10 μL of TaqMan 2× Universal PCR Master Mix and 1 μL of the specific TaqMan MicroRNA Assay (20×) containing probes for the miRNA of interest (Applied Biosystems, Darmstadt, Germany). The mixture was initially incubated at 95 °C for 10 min, followed by 40 cycles of 95 °C for 15 seconds and 60 °C for 60 seconds. Samples were run in duplicate and results were averaged RNU48 (U48) was used to normalize data. The relative miRNA expression was calculated using the 2−ΔCt method.

### Statistical analysis

Pearson’s Bivariate correlation was used to investigate statistical association between continuous and ordinal variable. GCS at admission disclosed strong associations with GOS at six months and survival (r = 0.572, p.007 and r = 0.604, p.004 respectively). Interestingly GCS was negatively associated to DAI grade (r = −0.541, p.011). Similarly, DAI grade was negatively associated to with survival (r = −0.616, p.003). GOS at six months was associated to survival (r = 0.856, p.0001), demonstrating that a better neurological outcome is more likely to be expressed to a long terminal surviving patient.

Data were expressed as the mean ± SD from at least three independent experiments. Statistical analysis was performed using Student’s t-test. The diagnostic evaluation of miR-21, miR-92 and miR-16 was performed through receiver operating characteristic (ROC) analysis. Areas under the curve (AUCs) were calculated. All statistical analyses were performed using Graph Pad Prism software (Graph Pad software). p < 0.05 was considered statistically significant.

### Approval, accordance and ethical statement

Institutional Ethics Committee of Sapienza University of Rome, expressed favorable opinion for this study. Clinical and radiological data reported are completely anonymized. Our work is coherent with the ethical standards proposed in the Helsinki declaration.
